# The automation of relevant trial registration screening for systematic review updates: an evaluation study on a large dataset of ClinicalTrials.gov registrations

**DOI:** 10.1186/s12874-021-01485-6

**Published:** 2021-12-18

**Authors:** Didi Surian, Florence T. Bourgeois, Adam G. Dunn

**Affiliations:** 1 Centre for Health Informatics, Australian Institute of Health Innovation, Macquarie University, Sydney, NSW Australia; 2 Computational Health Informatics Program, Boston Children’s Hospital, Boston, MA USA; 3 Department of Pediatrics, Harvard Medical School, Boston, MA USA; 4 The University of Sydney, Discipline of Biomedical Informatics and Digital Health, School of Medical Sciences, Faculty of Medicine and Health, Sydney, NSW 2006 Australia

**Keywords:** Systematic reviews, Trial registrations, Document similarity, Hierarchical clustering

## Abstract

**Background:**

Clinical trial registries can be used as sources of clinical evidence for systematic review synthesis and updating. Our aim was to evaluate methods for identifying clinical trial registrations that should be screened for inclusion in updates of published systematic reviews.

**Methods:**

A set of 4644 clinical trial registrations (ClinicalTrials.gov) included in 1089 systematic reviews (PubMed) were used to evaluate two methods (document similarity and hierarchical clustering) and representations (L2-normalised TF-IDF, Latent Dirichlet Allocation, and Doc2Vec) for ranking 163,501 completed clinical trials by relevance. Clinical trial registrations were ranked for each systematic review using seeding clinical trials, simulating how new relevant clinical trials could be automatically identified for an update. Performance was measured by the number of clinical trials that need to be screened to identify all relevant clinical trials.

**Results:**

Using the document similarity method with TF-IDF feature representation and Euclidean distance metric, all relevant clinical trials for half of the systematic reviews were identified after screening 99 trials (IQR 19 to 491). The best-performing hierarchical clustering was using Ward agglomerative clustering (with TF-IDF representation and Euclidean distance) and needed to screen 501 clinical trials (IQR 43 to 4363) to achieve the same result.

**Conclusion:**

An evaluation using a large set of mined links between published systematic reviews and clinical trial registrations showed that document similarity outperformed hierarchical clustering for identifying relevant clinical trials to include in systematic review updates.

**Supplementary Information:**

The online version contains supplementary material available at 10.1186/s12874-021-01485-6.

## Background

Systematic reviews provide efficient access to medical evidence for use in clinical decision-making and practice guidelines and policy development [1–3]. New evidence is accumulating at unprecedented rates in many domains, requiring systematic reviews to be almost continuously updated to remain current [4]. However, only around a third of reviews are updated within 2 years of being published, and the median update time is more than 5 years [5–7]. Even in application domains where systematic reviews are published frequently, it takes a median of 1.5 years from trial publication to inclusion in a first systematic review [8].

The reason for these delays is that performing systematic reviews is resource-intensive and efforts are inefficiently allocated. Systematic reviews take on average 881 person-hours across 463 days to complete [9]. In many cases, the processes and data used to perform a review are inaccessible or not provided in a format that could be applied to avoid re-work [10]. When systematic reviews are updated, few lead to a substantive change in conclusion [11, 12], suggesting that the process could be improved by prioritizing systematic reviews where the accumulation of new evidence indicates a higher likelihood of a revised conclusion.

Clinical study registries offer an opportunity to improve the efficiency of systematic review synthesis and updating but do not yet support informatics tools for this purpose. Registries have already been leveraged for a range of activities to improve the integrity and transparency of clinical trials, including reducing biases associated with trial non-publication and selective reporting practices [13–16]. Trial registries also have the potential to support systematic review processes by enabling a system to monitor ongoing trial activity and signal areas with higher rates of emerging evidence. This approach would depend on methods to accurately identify which clinical trial registrations were relevant to specific systematic review topics.

The aim of this study was to evaluate a new method for identifying and ranking clinical trial registrations most relevant to a systematic review update. To do this, we made use of a large database of mined links between published systematic reviews and the registrations of included trials.

## Methods

### Study data

The study data consisted of a set of published systematic reviews and sets of links to registered clinical trials included in the review. We retrieved systematic reviews available on PubMed on 21 October 2019 and published after 1 January 2007, using title keywords “systematic review” or “meta-analysis” in the title, and extracted the title, abstract, authors, publication date, PubMed ID, and Digital Object Identifier (DOI). The approach follows the methods used to populate a database of systematic reviews with mined links [17].

**Fig. 1:**
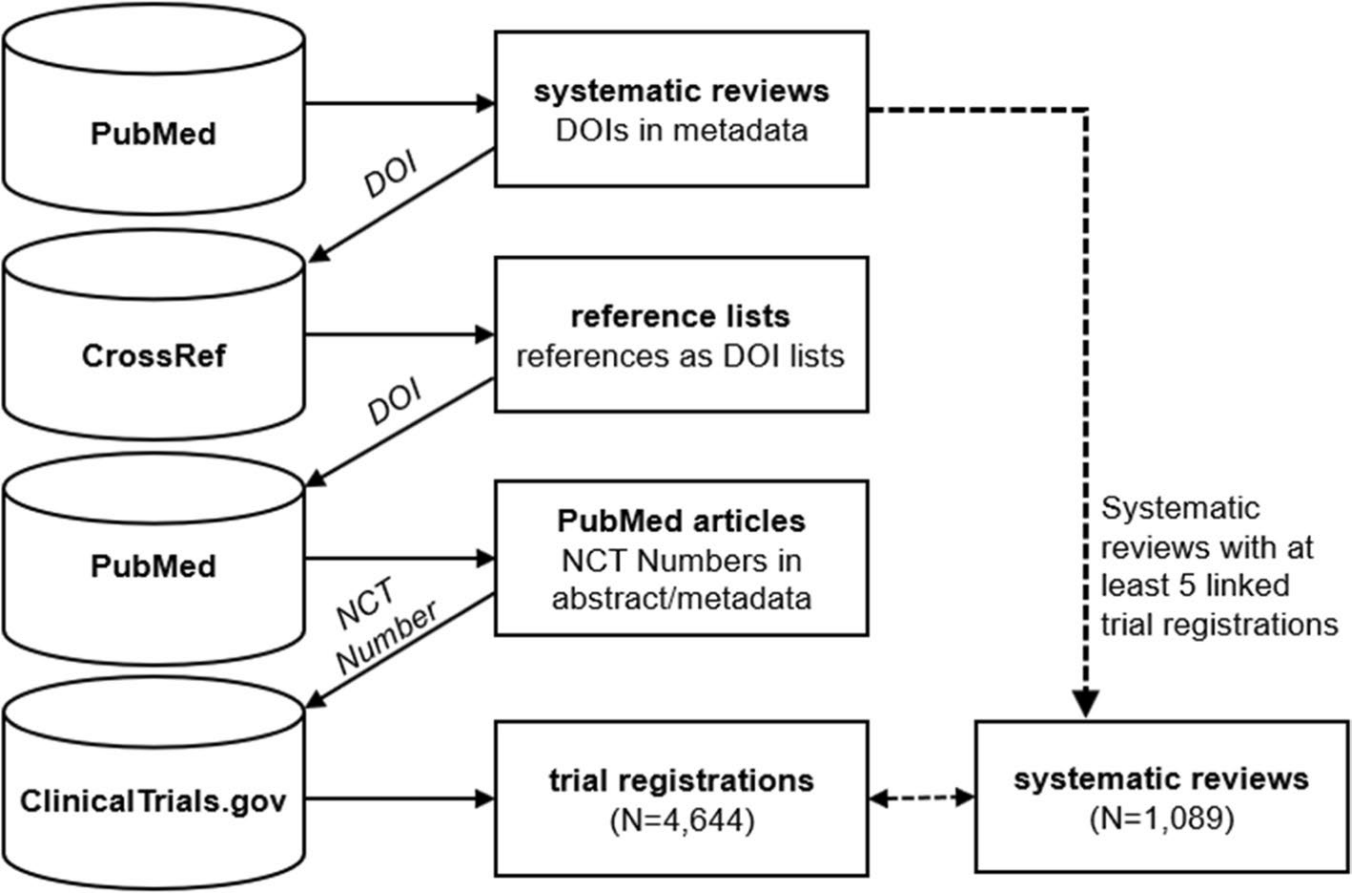
Construction of the dataset using PubMed, CrossRef, and ClinicalTrials.gov.

Systematic review DOIs identified on PubMed were then used to query CrossRef to identify and extract reference lists associated with the systematic review article, where they were made available through the Initiative for Open Citations. These lists of DOIs representing the articles cited by the systematic review were then searched for on PubMed, where the presence of an NCT Number in the article abstract or metadata was used to determine if there was a corresponding registration for a completed trial on ClinicalTrials.gov (Fig. 1). This process assumes that the systematic review reference lists include studies used in the evidence synthesis and that the PubMed entries for other cited articles generally do not have abstract or metadata links to ClinicalTrials.gov registrations. Prior analyses involving manual validation of links between systematic review reference lists and trial registries suggest that this approach is highly specific (nearly all links are true positives) but can miss studies (many false negatives) that were not linked by metadata to registrations on ClinicalTrials.gov [18–20].

For the evaluation and analysis, we included only those systematic reviews that had at least five links to trial registrations. For each of the trials identified via the above process, we extracted the text from the brief and official titles, summary, and detailed description in the ClinicalTrials.gov record, and used this text as the features in the experiments described below.

### Feature representation

We treated each trial registration as a single document and performed standard text pre-processing steps. Each word was converted to lowercase and stop words, punctuations, and names of the months removed. We also excluded numbers, unless the number was part of a combination of characters and numbers (e.g., H1N1, Omega-3). The Porter stemmer was then applied to convert each word to its word stem [21]. To reduce the number of uninformative common and very rare words, we applied typical thresholds to only include words that had more than one character and appeared in more than two trial registrations or less than 95% of all trial registrations. We chose not to map text to medical concepts based on the results of a previous study, which indicated better performance using words compared to medical concepts for linking ClinicalTrials.gov and abstracts of trial reports [20].

Each trial registration was represented by one of three feature representations based on the extracted words. First, we used the Term Frequency-Inverse Document Frequency (TF-IDF). When calculating the TF-IDF score, we used the logarithm of the term frequency. Each trial registration was represented by a vector of the L2-normalised TF-IDF score of a word feature with a dimension equal to the size of the vocabulary. Second, we used Latent Dirichlet Allocation (LDA) to represent a trial registration as a vector of topic distribution. LDA method was first introduced by Blei et al. [22] to extract latent structures, or topics, in a corpus of text documents, where a document is represented by a distribution of topics and a topic is represented by a vector of word probabilities. In LDA, each document is represented by a bag of words, where the order of words is ignored. We used the implementation of LDA from *Gensim* for python programming language with a standard parameter setting [23, 24], and tested with 50, 100, 150, and 200 topics. Third, we used Paragraph Vector, or Doc2Vec, to represent a trial registration as a vector representation. Doc2Vec was proposed by Le and Mikolov [25], as an unsupervised method to learn a vector representation for text. We also used the implementation of Doc2Vec from *Gensim* for python programming language with distributed memory setting that preserves the word order in a document and tested with 50, 100, 150, and 200 vector dimensions. The selection of parameters for pre-processing and feature representation was based on our previous experience with similar corpora.

### Experiment design and performance measures

For each systematic review, we ordered the trial registrations by their completion dates and used 80% of older trial registrations as our training (seeding) set and the remaining 20% as a test set. The aim was to mimic how a systematic review would be updated to include results from newer trials, with the seeding set representing trials in an existing systematic review and the test set new trials that should be screened for inclusion in an update. The number of trial registrations per systematic review ranged from 4 to 55 in the seeding set and 1 to 13 in the test set.

For each systematic review and starting from a seeding set of between 4 to 55 included trials, the task was then to rank all other trial registrations in ClinicalTrials.gov such that the 1 to 13 in the test set were ranked as high as possible. We evaluated the performance of our methods by measuring the number of trial registrations that needed to be screened to capture all test set trial registrations. This was reported as the median number of trial registrations screened to achieve 100% recall across each of the systematic reviews. We also calculated the median recall (by aggregating the recall values for all systematic reviews and taking the median) after screening a given number of trial registrations for each systematic review. Using the median recall, we determined which method resulted in the best trial ranking. We also evaluated whether the size of the seeding set (i.e., the number of trials already included in the review) contributed to the performance of each method.

### Document similarity

For each systematic review, we calculated the Euclidean distance between each of the trial registrations in the seeding set and the set of test trials as well as any trial registration in the dataset with completion dates after the systematic review’s publication date. The Euclidean distance uses the representation of each document (the weights associated with each of the words in the vocabulary) and is given by the square root of the sum of the differences between the corresponding weights in the two documents. As each trial registration in the dataset has different distance values to the trial registrations in the seeding set, we considered the smallest distance to any trial registration in the seeding set as the distance between a trial registration and the systematic review to which it might belong.

Trial registrations were then ranked based on their distance values in ascending order and the retrieval process started from the trial registration with the smallest distance. This is similar to the ordered representation that would be used in tools that rank articles in search results from bibliographic databases to support screening [26–28].

### Hierarchical agglomerative clustering

**Fig. 2:**
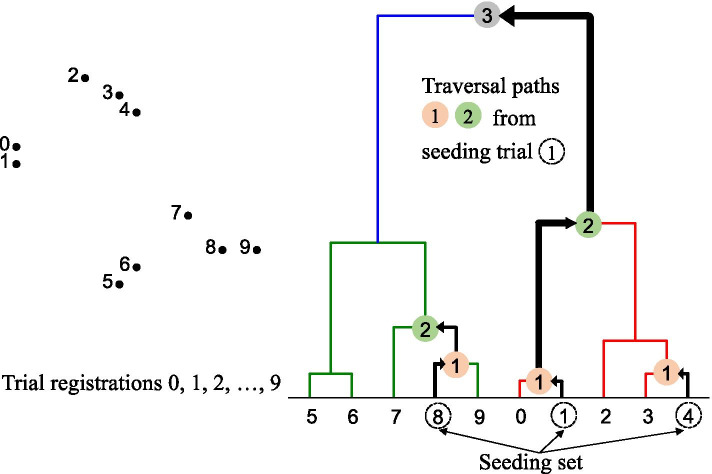
Illustration of the hierarchical agglomerative clustering method (left) and traversal on the resulting dendrogram (right)

Clustering methods seek to place items into groups (called clusters) and hard clustering methods seek to place items such that no items belong to more than one cluster. Agglomerative clustering methods are a type of hard clustering that start with each item in its own cluster and iteratively combines clusters until an optimal clustering is found. The hierarchical agglomerative clustering method groups the two most similar data points into the same cluster and then iteratively groups two newly formed clusters until only one cluster remains. It can be represented as a dendrogram (a hierarchical representation) where trial registrations are leaf nodes and groups of trial registrations are represented under other nodes in the hierarchy (Fig. 2).

To compute the distance between clusters, we used single-linkage and Ward agglomerative methods and applied them to the 163,501 trial registrations in the dataset. The single-linkage agglomerative method takes the smallest distance between two points in two clusters as the distance between clusters, while the Ward agglomerative method uses the Ward variance minimization algorithm to compute the distance between two clusters [29]. We used the *fastcluster* library for python programming language to perform the hierarchical clustering on the trial registrations [30].

Given a hierarchical representation of the trials, the traversal starts with the seeding set of example trials and traverses up the hierarchy in steps, recommending additional trials at the leaf nodes during the traversal up to the root node (Fig. 2). The trials were then ranked in the order they were encountered during the traversal. For example, in Fig. 2, the trial ranking would be 0, 3, 9, 2, 7, 6, and 5. As with the document similarity approach, we ignored any trial registration that was not in the test set or published after the publication date of the systematic review to simulate a systematic review update scenario.

### Verified trial registrations

The dataset used in the experiments described above is highly specific but imperfect set of examples of included trials. To examine the performance of the methods on a more realistic set of examples as a form of error analysis, we selected ten systematic reviews that were not included in the dataset above and manually verified the set of included trials with registrations on ClinicalTrials.gov. Systematic reviews were selected by searching for systematic reviews that examined novel therapeutics approved by the FDA in 2019.

Trial registrations were identified by identifying the set of included trials from the full text of the systematic review and then following a standard approach for identifying registrations linked to the published results [19]. For each included trial, this includes checking for NCT Numbers in the published trial article abstract, metadata, and full text, and searching ClinicalTrials.gov for the intervention and comparing trial design features for returned results where no information about trial registration is included in the text of the article.

After splitting the included trial registrations into seeding and test sets for the ten systematic reviews, the number of verified trial registrations ranged from 5 to 12 in the seeding set and from 1 to 2 in the test set. These ten systematic reviews with verified trial registrations were used to examine the performance of the methods in a more realistic scenario and to examine where and why the methods perform poorly for some systematic reviews.

## Results

The process produced a final dataset of 1089 systematic reviews linked to 4644 unique trials (from a total of 163,501 completed trials registered on ClinicalTrials.gov). The number of links per systematic review varied from 5 to 69 and some trials were included in more than one systematic review. The average number of links from trial registrations to systematic reviews was 2 (ranging from 1 to 53). We extracted and stored a local copy of the data on 21 October 2019 for use in the analysis.

There were 100,000 unique words in the vocabulary extracted from all trial registrations after the text pre-processing step. For each systematic review, the number of words shared between the trials in the seeding set and the trials in the test set ranged from 29 to 1955.

### Tests using mined trial registration links

For the hierarchical clustering method, the combination of TF-IDF feature representation, the Euclidean distance metric, and the Ward agglomerative method produced the best performance (Table 1). In the best performing combination, 100% recall was achieved for half of the 1089 systematic reviews after screening 501 trial registrations per review. The single-linkage agglomerative method consistently produced lower performance than the Ward agglomerative method on any feature representations. The Doc2Vec feature representation gave the worst performance compared with the other feature representations.

**Table 1:** The median number of trial registrations to be screened to achieve 100% recall

Model	Median [IQR]
**Hierarchical clustering**	
TF-IDF, Ward, Euclidean	**501 [43–4363]**
Single-linkage, Euclidean	90,725 [31070–132,615]
LDA, 50 topics, Ward, Euclidean	6352 [986–86,990]
100 topics	4381 [465–91,954]
150 topics	4653 [687–77,875]
200 topics	4453 [425–70,500]
LDA, 50 topics, Single-linkage, Euclidean	112,858 [71482–136,676]
100 topics	115,957 [67384–137,334]
150 topics	113,794 [76429–139,497]
200 topics	124,259 [89399–146,225]
Doc2Vec, 50-dimensional vector, Ward, Euclidean	2256 [198–23,926]
100-dimensional vector	2432 [176–26,889]
150-dimensional vector	3850 [238–78,118]
200-dimensional vector	5113 [231–70,483]
Doc2Vec, 50 vector dimension, Single-linkage, Euclidean	125,000 [84772–150,519]
100-dimensional vector	127,604 [91965–150,958]
150-dimensional vector	128,801 [89896–151,288]
200-dimensional vector	128,978 [89398–151,171]
**Document similarity**	
TF-IDF, Euclidean	**99 [19–491]**
LDA, 50 topics, Euclidean	1287 [271–4968]
100 topics	842 [134–3776]
150 topics	793 [123–4268]
200 topics	887 [116–5417]
Doc2Vec, 50-dimensional vector, Euclidean	18,501 [1970–51,495]
100-dimensional vector	33,968 [7898–68,806]
150-dimensional vector	41,116 [12036–77,218]
200-dimensional vector	43,879 [13791–82,388]

**Fig. 3:**
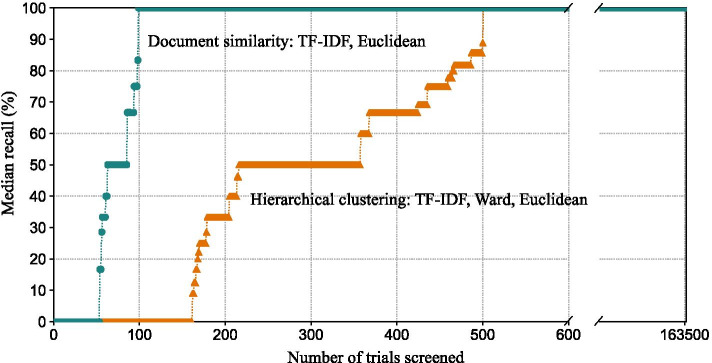
The median recall for 1089 systematic reviews after screening a given number of trial registrations

The combination of TF-IDF feature representation and the Euclidean distance metric gave the best performance for the document similarity method. 100% recall was achieved for half of the systematic reviews after screening 99 trial registrations per review. This represents a decrease of 80.2% relative to the best performing hierarchical clustering method and the difference in performance is consistently higher after screening any number of trial registrations (Fig. 3). The Doc2Vec feature representation again had the lowest performance.

**Fig. 4:**
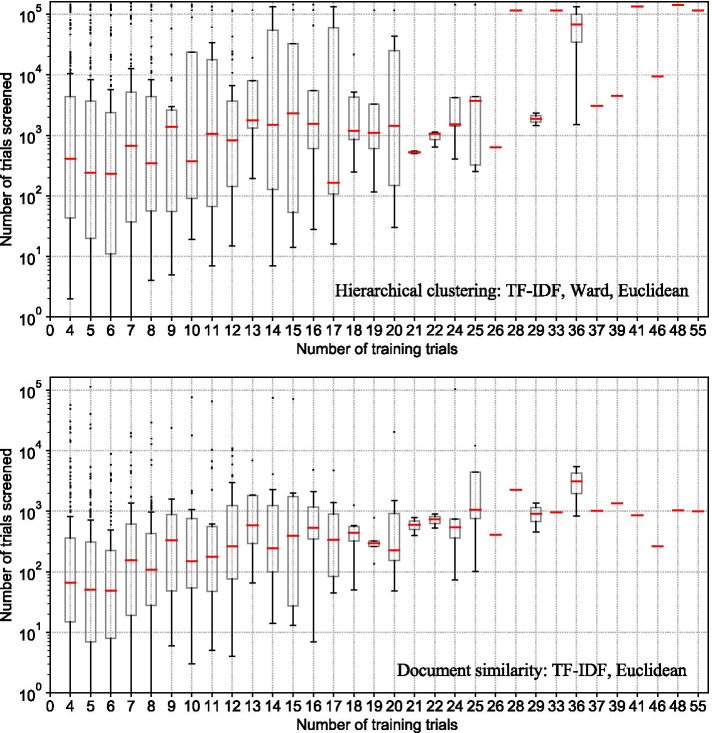
Effect of seeding set size to the number of trials screened to achieve 95% recall

We analyzed whether the number of trial registrations in the seeding set was associated with the number needed to screen. Measuring how many trials needed to be screened to achieve at least 95% recall in the test set relative to the number of trials in the seeding set, there is evidence of an increase in the number that needs to be screened (Fig. 4). The results suggest that where systematic reviews are broader in scope, more trials need to be screened for inclusion. The use of seeding trials that may be distant from each other in the hierarchy (hierarchical clustering method) as well as defining the minimum distance to any trial in the seeding set rather than to the centroid of the seeding set (document similarity method) appears to only partially limit the need to screen more trial registrations as the number of included trials increases.

Full results across the set of feature representations and distance metrics for both document similarity and hierarchical clustering methods are provided in the Supplementary Material.

### Tests using verified trial registrations

In the set of ten verified systematic reviews, the best performing document similarity method outperformed the hierarchical clustering method. For hierarchical clustering with the combination of TF-IDF feature representation, Euclidean distance metric, and Ward agglomerative method, the median number of trials that needed to be screened to reach 100% recall was 31 (IQR 6-226) and ranged from 4 to 2808. For the document similarity approach, the combination of TF-IDF feature representation and Euclidean distance metric, the median number of trials that needed to be screened to reach 100% recall was 13 (IQR 5-31) and ranged from 1 to 54.

The methods performed better when evaluated on the 10 systematic reviews with manually verified links compared to the set of 1089 systematic reviews with mined links. The manually verified set may reflect a more realistic scenario for screening trials in individual systematic review updates and the mined set may better reflect use cases related to signals that are used to prioritize systematic reviews for updates.

## Discussion

In this evaluation study, we used a large dataset of systematic reviews linked to trial registrations to test different representations and methods to support automated processes for screening trials for inclusion in systematic review updates. We found that a relatively simple document similarity approach outperformed the more sophisticated method using hierarchical clustering and traversal. The results indicate that it may be feasible to develop tools to identify a relatively small number of trials that need to be screened for inclusion in systematic review updates. However, it is not yet possible to automatically and directly allocate new trials for inclusion in systematic review updates or predict the availability of new evidence without manual effort.

Other studies have examined methods for reducing workload in identifying and screening relevant articles for systematic review topics, and many have used content from abstracts and full-text articles [28, 31–33]. Our work differs from these previous studies in two important ways. First, our methods are based on screening trial registrations rather than published trial report abstracts, allowing for earlier identification of potentially relevant trials. Second, instead of using supervised machine learning methods to classify articles by training on positive and negative labels manually assigned to articles for a small number of reviews, our method uses mined positive labels of included trial registrations, enabling analysis and development on a much larger and more general set of systematic reviews.

**Fig. 5:**
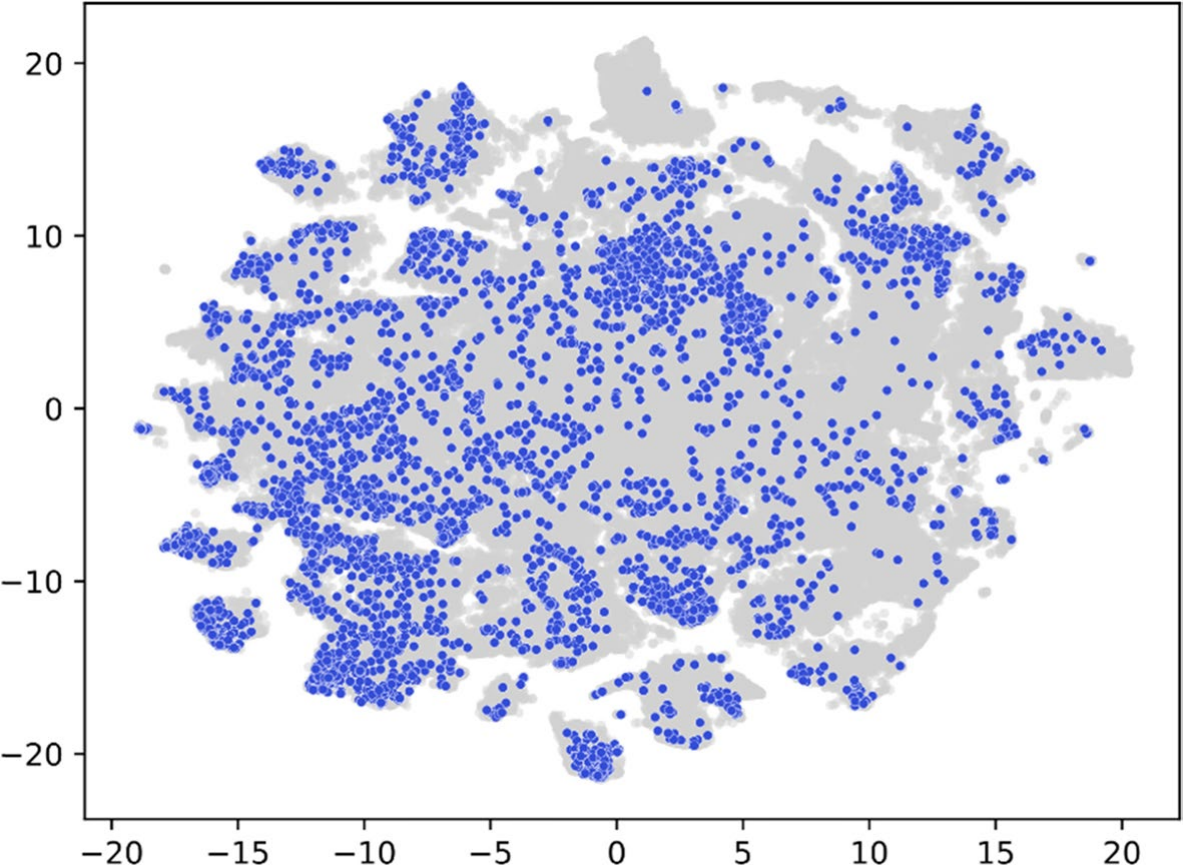
The t-SNE visualization of the evaluated 4644 trials (blue) and the other ClinicalTrials.gov trials (grey)

In a previous study, we identified relevant trials using matrix factorization to incorporate information from the texts of trial registrations and the links between systematic reviews and trial registrations [34]. The methods tested here improve the performance in two ways. First, the methods tested in this study do not depend on labeled examples for training as in the matrix factorization approach. Second, the matrix factorization method was limited by a known problem associated with recommender systems—it worked best when tested on systematic reviews with similar topics as the trained data and struggled when links were sparsely distributed. In this study, trials were broadly distributed across diseases and interventions, with more than six times as many systematic reviews as in the previous study (1089 compared to 179). Figure 5 illustrates the breadth of topics covered in the set of systematic reviews using t-SNE [35], a visualization method where similar trials are drawn close together.

This work has several implications for evidence synthesis. Existing tools supporting trial screening for systematic reviews are designed to reduce workloads after a systematic review has been designed and bibliographic searches conducted. Our proposed tool differs in that it enables automatic identification of relevant trials without repeating searches. By relying on registrations instead of publications, the approach can serve to alert systematic reviewers when a potentially relevant trial is registered, completed, and then reported. Such a tool would suit living systematic reviews, where updates are performed whenever new evidence is detected. In practical terms, a tool based on this approach could alert a systematic reviewer to a potentially relevant trial as it is registered, it could be screened for inclusion, and further alerts could be sent to the reviewer as the status of the trial changes to complete, or when results are first posted. Another tool that could be developed is one that supports the prioritization of systematic review updates, by providing early indicators of the number and sizes of new and relevant trials that suggest when a systematic review update is warranted and flagging published systematic reviews that are likely out of date.

### Limitations

This study has several limitations. First, we included all completed trial registrations available through linked reference lists in a large set of systematic reviews. A small number of these may represent trials that were cited for other reasons but were not included studies in the systematic review. We are also likely to have missed registrations of included trial registrations that were not linked via metadata to ClinicalTrials.gov [18–20]. When we tested the methods on a small set of manually verified systematic reviews, we observed a performance improvement, suggesting that the approach is better suited to uses cases where complete records of included studies are available. Second, we only tested some feature representations, distance measures, and agglomeration methods. Other combinations of representations, agglomeration methods, and distance metrics could have been used, and we may not have found the optimal combination. Newer representations such as pre-trained language models that embed context information may also lead to improvements in performance when combined with certain distance metrics.

### Future directions

Given the performance of the methods in relatively realistic scenarios—splitting included trials from systematic reviews chronologically and identifying recently published trials using information from trials published earlier—the next logical step would be to test the methods as a tool for use in new systematic review updates. The tool would need to be tested in comparison to existing standards for searching and screening in systematic review updates and an appropriate study design would randomize tool use across two groups and compare workload, completeness of included studies, and systematic review conclusions. An experiment of this size would require cooperation across groups of systematic review teams and a substantial investment of time. Other future directions might include more sophisticated machine learning methods for automatically identifying trial registrations, perhaps by coupling with information extracted from the abstracts and metadata of published trial articles.

## Conclusions

Clinical trial registries can be leveraged to support new methods to signal when a systematic review update is warranted and to efficiently perform trial screening tasks. In this evaluation using a large set of links between systematic reviews and trial registrations, we demonstrate methods that start from a set of included trials and identify trials for further screening and inclusion in systematic review updates. Tools based on these methods could be used to improve how we update systematic reviews by helping to monitor trial registrations relative to published systematic reviews to signal when an update is warranted.

## Supplementary Information

**Additional file 1.**

## Data Availability

The datasets generated and/or analyzed during the current study are available from https://ClinicalTrials.gov and can be reconstructed from mined links available at https://es3-bidh.sydney.edu.au/.

## Abbreviations

TF-IDF: Term Frequency-Inverse Document Frequency
IQR: Interquartile Range
DOI: Digital Object Identifier
NCT Number: National Clinical Trial Number
LDA: Latent Dirichlet Allocation
FDA: Food and Drug Administration
t-SNE: t-distributed Stochastic Neighbor Embedding
