# Supplementary material for: The automation of relevant trial registration screening for systematic review updates: an evaluation study on a large dataset of ClinicalTrials.gov registrations
Source: BMC Med Res Methodol. 2021 Dec 18;21:281. doi: 10.1186/s12874-021-01485-6 (PMC8684229; doi:10.1186/s12874-021-01485-6)
Supplement: Supplementary file 1 — Additional file 1. [file 12874_2021_1485_MOESM1_ESM.pdf]

Combination	Recall	Q1	Q2	Q3	IQR	Median
Docsim, Tfidf, euclidean	0.8	19	98	469	450	98
Hierarchy, Tfidf, ward, euclidean	0.8	43	465	4247	4204	465
Hierarchy, Tfidf, single, euclidean	0.8	31057	89202	132100	101043	89202
Hierarchy, Tfidf, single, cosine	0.8	31057	89202	132100	101043	89202
Docsim, LDA_50, euclidean	0.8	261	1269	4751	4490	1269
Hierarchy, LDA_50, ward, euclidean	0.8	975	6120	82477	81502	6120
Hierarchy, LDA_50, single, euclidean	0.8	71349	112354	136553	65204	112354
Hierarchy, LDA_50, single, cosine	0.8	78157	112376	140663	62506	112376
Docsim, LDA_100, euclidean	0.8	131	824	3588	3457	824
Hierarchy, LDA_100, ward, euclidean	0.8	465	4082	84298	83833	4082
Hierarchy, LDA_100, single, euclidean	0.8	67269	115353	137230	69961	115353
Hierarchy, LDA_100, single, cosine	0.8	68010	111069	136727	68717	111069
Docsim, LDA_150, euclidean	0.8	123	775	4006	3883	775
Hierarchy, LDA_150, ward, euclidean	0.8	685	4344	63684	62999	4344
Hierarchy, LDA_150, single, euclidean	0.8	75312	113107	139404	64092	113107
Hierarchy, LDA_150, single, cosine	0.8	77657	113190	134609	56952	113190
Docsim, LDA_200, euclidean	0.8	112	875	5139	5027	875
Hierarchy, LDA_200, ward, euclidean	0.8	421	4249	69976	69555	4249
Hierarchy, LDA_200, single, euclidean	0.8	89399	123593	146048	56649	123593
Hierarchy, LDA_200, single, cosine	0.8	79481	116807	142796	63315	116807
Docsim, D2V_50, euclidean	0.8	1970	18190	50763	48793	18190
Hierarchy, D2V_50, ward, euclidean	0.8	198	2172	21589	21391	2172
Hierarchy, D2V_50, single, euclidean	0.8	84719	124358	149750	65031	124358
Hierarchy, D2V_50, single, cosine	0.8	80451	118633	140232	59781	118633
Docsim, D2V_100, euclidean	0.8	7898	33246	67012	59114	33246
Hierarchy, D2V_100, ward, euclidean	0.8	176	2319	22261	22085	2319
Hierarchy, D2V_100, single, euclidean	0.8	91922	127509	150641	58719	127509
Hierarchy, D2V_100, single, cosine	0.8	76386	119083	143503	67117	119083
Docsim, D2V_150, euclidean	0.8	11992	39419	76665	64673	39419

Hierarchy, D2V_150, ward, euclidean	0.8	238	3490	54271	54033	3490
Hierarchy, D2V_150, single, euclidean	0.8	89557	128630	151288	61731	128630
Hierarchy, D2V_150, single, cosine	0.8	70374	114253	145977	75603	114253
Docsim, D2V_200, euclidean	0.8	13643	42270	81841	68198	42270
Hierarchy, D2V_200, ward, euclidean	0.8	224	4491	63873	63649	4491
Hierarchy, D2V_200, single, euclidean	0.8	89090	128967	151171	62081	128967
Hierarchy, D2V_200, single, cosine	0.8	72446	120180	143648	71202	120180
Docsim, Tfidf, euclidean	0.85	19	99	489	470	99
Hierarchy, Tfidf, ward, euclidean	0.85	43	487	4284	4241	487
Hierarchy, Tfidf, single, euclidean	0.85	31068	90725	132368	101300	90725
Hierarchy, Tfidf, single, cosine	0.85	31068	90725	132368	101300	90725
Docsim, LDA_50, euclidean	0.85	271	1287	4879	4608	1287
Hierarchy, LDA_50, ward, euclidean	0.85	986	6286	85253	84267	6286
Hierarchy, LDA_50, single, euclidean	0.85	71482	112362	136556	65074	112362
Hierarchy, LDA_50, single, cosine	0.85	78178	112775	141246	63068	112775
Docsim, LDA_100, euclidean	0.85	134	842	3717	3583	842
Hierarchy, LDA_100, ward, euclidean	0.85	465	4314	89772	89307	4314
Hierarchy, LDA_100, single, euclidean	0.85	67384	115957	137230	69846	115957
Hierarchy, LDA_100, single, cosine	0.85	68692	111826	136728	68036	111826
Docsim, LDA_150, euclidean	0.85	123	789	4166	4043	789
Hierarchy, LDA_150, ward, euclidean	0.85	687	4626	74844	74157	4626
Hierarchy, LDA_150, single, euclidean	0.85	76377	113114	139404	63027	113114
Hierarchy, LDA_150, single, cosine	0.85	78445	113459	134609	56164	113459
Docsim, LDA_200, euclidean	0.85	116	885	5386	5270	885
Hierarchy, LDA_200, ward, euclidean	0.85	421	4363	70364	69943	4363
Hierarchy, LDA_200, single, euclidean	0.85	89399	123869	146048	56649	123869
Hierarchy, LDA_200, single, cosine	0.85	79481	117194	142796	63315	117194
Docsim, D2V_50, euclidean	0.85	1970	18501	51276	49306	18501
Hierarchy, D2V_50, ward, euclidean	0.85	198	2256	23721	23523	2256
Hierarchy, D2V_50, single, euclidean	0.85	84772	124537	149855	65083	124537

Hierarchy, D2V_50, single, cosine	0.85	80841	118633	140232	59391	118633
Docsim, D2V_100, euclidean	0.85	7898	33968	67928	60030	33968
Hierarchy, D2V_100, ward, euclidean	0.85	176	2388	24724	24548	2388
Hierarchy, D2V_100, single, euclidean	0.85	91965	127604	150720	58755	127604
Hierarchy, D2V_100, single, cosine	0.85	76386	119083	143503	67117	119083
Docsim, D2V_150, euclidean	0.85	12036	41116	77002	64966	41116
Hierarchy, D2V_150, ward, euclidean	0.85	238	3822	64162	63924	3822
Hierarchy, D2V_150, single, euclidean	0.85	89896	128801	151288	61392	128801
Hierarchy, D2V_150, single, cosine	0.85	71542	114253	146031	74489	114253
Docsim, D2V_200, euclidean	0.85	13791	43879	82010	68219	43879
Hierarchy, D2V_200, ward, euclidean	0.85	231	5075	70482	70251	5075
Hierarchy, D2V_200, single, euclidean	0.85	89398	128978	151171	61773	128978
Hierarchy, D2V_200, single, cosine	0.85	72446	120180	143648	71202	120180
Docsim, Tfidf, euclidean	0.9	19	99	491	472	99
Hierarchy, Tfidf, ward, euclidean	0.9	43	501	4284	4241	501
Hierarchy, Tfidf, single, euclidean	0.9	31068	90725	132612	101544	90725
Hierarchy, Tfidf, single, cosine	0.9	31068	90725	132612	101544	90725
Docsim, LDA_50, euclidean	0.9	271	1287	4961	4690	1287
Hierarchy, LDA_50, ward, euclidean	0.9	986	6352	86990	86004	6352
Hierarchy, LDA_50, single, euclidean	0.9	71482	112842	136584	65102	112842
Hierarchy, LDA_50, single, cosine	0.9	78178	113211	141246	63068	113211
Docsim, LDA_100, euclidean	0.9	134	842	3749	3615	842
Hierarchy, LDA_100, ward, euclidean	0.9	465	4314	91056	90591	4314
Hierarchy, LDA_100, single, euclidean	0.9	67384	115957	137334	69950	115957
Hierarchy, LDA_100, single, cosine	0.9	68692	112251	136730	68038	112251
Docsim, LDA_150, euclidean	0.9	123	789	4195	4072	789
Hierarchy, LDA_150, ward, euclidean	0.9	687	4651	76248	75561	4651
Hierarchy, LDA_150, single, euclidean	0.9	76429	113646	139404	62975	113646
Hierarchy, LDA_150, single, cosine	0.9	78445	113463	134673	56228	113463
Docsim, LDA_200, euclidean	0.9	116	887	5392	5276	887

Hierarchy, LDA_200, ward, euclidean	0.9	425	4453	70364	69939	4453
Hierarchy, LDA_200, single, euclidean	0.9	89399	124259	146206	56807	124259
Hierarchy, LDA_200, single, cosine	0.9	79481	117195	142835	63354	117195
Docsim, D2V_50, euclidean	0.9	1970	18501	51495	49525	18501
Hierarchy, D2V_50, ward, euclidean	0.9	198	2256	23722	23524	2256
Hierarchy, D2V_50, single, euclidean	0.9	84772	125000	150166	65394	125000
Hierarchy, D2V_50, single, cosine	0.9	80841	118633	140232	59391	118633
Docsim, D2V_100, euclidean	0.9	7898	33968	68454	60556	33968
Hierarchy, D2V_100, ward, euclidean	0.9	176	2426	25777	25601	2426
Hierarchy, D2V_100, single, euclidean	0.9	91965	127604	150958	58993	127604
Hierarchy, D2V_100, single, cosine	0.9	76386	119665	143503	67117	119665
Docsim, D2V_150, euclidean	0.9	12036	41116	77178	65142	41116
Hierarchy, D2V_150, ward, euclidean	0.9	238	3850	70729	70491	3850
Hierarchy, D2V_150, single, euclidean	0.9	89896	128801	151288	61392	128801
Hierarchy, D2V_150, single, cosine	0.9	71542	114258	146092	74550	114258
Docsim, D2V_200, euclidean	0.9	13791	43879	82044	68253	43879
Hierarchy, D2V_200, ward, euclidean	0.9	231	5075	70483	70252	5075
Hierarchy, D2V_200, single, euclidean	0.9	89398	128978	151171	61773	128978
Hierarchy, D2V_200, single, cosine	0.9	72446	120195	143648	71202	120195
Docsim, Tfidf, euclidean	0.95	19	99	491	472	99
Hierarchy, Tfidf, ward, euclidean	0.95	43	501	4363	4320	501
Hierarchy, Tfidf, single, euclidean	0.95	31070	90725	132615	101545	90725
Hierarchy, Tfidf, single, cosine	0.95	31070	90725	132615	101545	90725
Docsim, LDA_50, euclidean	0.95	271	1287	4968	4697	1287
Hierarchy, LDA_50, ward, euclidean	0.95	986	6352	86990	86004	6352
Hierarchy, LDA_50, single, euclidean	0.95	71482	112858	136676	65194	112858
Hierarchy, LDA_50, single, cosine	0.95	78178	113709	141246	63068	113709
Docsim, LDA_100, euclidean	0.95	134	842	3776	3642	842
Hierarchy, LDA_100, ward, euclidean	0.95	465	4381	91954	91489	4381
Hierarchy, LDA_100, single, euclidean	0.95	67384	115957	137334	69950	115957

Hierarchy, LDA_100, single, cosine	0.95	68692	112251	136730	68038	112251
Docsim, LDA_150, euclidean	0.95	123	793	4268	4145	793
Hierarchy, LDA_150, ward, euclidean	0.95	687	4653	77875	77188	4653
Hierarchy, LDA_150, single, euclidean	0.95	76429	113794	139497	63068	113794
Hierarchy, LDA_150, single, cosine	0.95	78445	113465	134687	56242	113465
Docsim, LDA_200, euclidean	0.95	116	887	5417	5301	887
Hierarchy, LDA_200, ward, euclidean	0.95	425	4453	70500	70075	4453
Hierarchy, LDA_200, single, euclidean	0.95	89399	124259	146225	56826	124259
Hierarchy, LDA_200, single, cosine	0.95	79481	117196	142836	63355	117196
Docsim, D2V_50, euclidean	0.95	1970	18501	51495	49525	18501
Hierarchy, D2V_50, ward, euclidean	0.95	198	2256	23926	23728	2256
Hierarchy, D2V_50, single, euclidean	0.95	84772	125000	150519	65747	125000
Hierarchy, D2V_50, single, cosine	0.95	81352	118633	140232	58880	118633
Docsim, D2V_100, euclidean	0.95	7898	33968	68806	60908	33968
Hierarchy, D2V_100, ward, euclidean	0.95	176	2432	26889	26713	2432
Hierarchy, D2V_100, single, euclidean	0.95	91965	127604	150958	58993	127604
Hierarchy, D2V_100, single, cosine	0.95	76386	119665	143503	67117	119665
Docsim, D2V_150, euclidean	0.95	12036	41116	77218	65182	41116
Hierarchy, D2V_150, ward, euclidean	0.95	238	3850	78118	77880	3850
Hierarchy, D2V_150, single, euclidean	0.95	89896	128801	151288	61392	128801
Hierarchy, D2V_150, single, cosine	0.95	71542	114258	146092	74550	114258
Docsim, D2V_200, euclidean	0.95	13791	43879	82388	68597	43879
Hierarchy, D2V_200, ward, euclidean	0.95	231	5113	70483	70252	5113
Hierarchy, D2V_200, single, euclidean	0.95	89398	128978	151171	61773	128978
Hierarchy, D2V_200, single, cosine	0.95	72446	120195	143841	71395	120195
Docsim, D2V_100, euclidean	1	7898	33968	68806	60908	33968
Docsim, D2V_150, euclidean	1	12036	41116	77218	65182	41116
Docsim, D2V_200, euclidean	1	13791	43879	82388	68597	43879
Docsim, D2V_50, euclidean	1	1970	18501	51495	49525	18501
Docsim, LDA_100, euclidean	1	134	842	3776	3642	842

Docsim, LDA_150, euclidean	1	123	793	4268	4145	793
Docsim, LDA_200, euclidean	1	116	887	5417	5301	887
Docsim, LDA_50, euclidean	1	271	1287	4968	4697	1287
Docsim, Tfidf, euclidean	1	19	99	491	472	99
Hierarchy, D2V_100, single, cosine	1	76386	119665	143503	67117	119665
Hierarchy, D2V_100, single, euclidean	1	91965	127604	150958	58993	127604
Hierarchy, D2V_100, ward, euclidean	1	176	2432	26889	26713	2432
Hierarchy, D2V_150, single, cosine	1	71542	114258	146092	74550	114258
Hierarchy, D2V_150, single, euclidean	1	89896	128801	151288	61392	128801
Hierarchy, D2V_150, ward, euclidean	1	238	3850	78118	77880	3850
Hierarchy, D2V_200, single, cosine	1	72446	120195	143841	71395	120195
Hierarchy, D2V_200, single, euclidean	1	89398	128978	151171	61773	128978
Hierarchy, D2V_200, ward, euclidean	1	231	5113	70483	70252	5113
Hierarchy, D2V_50, single, cosine	1	81352	118633	140232	58880	118633
Hierarchy, D2V_50, single, euclidean	1	84772	125000	150519	65747	125000
Hierarchy, D2V_50, ward, euclidean	1	198	2256	23926	23728	2256
Hierarchy, LDA_100, single, cosine	1	68692	112251	136730	68038	112251
Hierarchy, LDA_100, single, euclidean	1	67384	115957	137334	69950	115957
Hierarchy, LDA_100, ward, euclidean	1	465	4381	91954	91489	4381
Hierarchy, LDA_150, single, cosine	1	78445	113465	134687	56242	113465
Hierarchy, LDA_150, single, euclidean	1	76429	113794	139497	63068	113794
Hierarchy, LDA_150, ward, euclidean	1	687	4653	77875	77188	4653
Hierarchy, LDA_200, single, cosine	1	79481	117196	142836	63355	117196
Hierarchy, LDA_200, single, euclidean	1	89399	124259	146225	56826	124259
Hierarchy, LDA_200, ward, euclidean	1	425	4453	70500	70075	4453
Hierarchy, LDA_50, single, cosine	1	78178	113709	141246	63068	113709
Hierarchy, LDA_50, single, euclidean	1	71482	112858	136676	65194	112858
Hierarchy, LDA_50, ward, euclidean	1	986	6352	86990	86004	6352
Hierarchy, Tfidf, single, cosine	1	31070	90725	132615	101545	90725
Hierarchy, Tfidf, single, euclidean	1	31070	90725	132615	101545	90725

Hierarchy, Tfidf, ward, euclidean	1	43	501	4363	4320	501
